# Anti-HSV-1 agents: an update

**DOI:** 10.3389/fphar.2024.1451083

**Published:** 2025-01-21

**Authors:** Wenwen Lv, Lei Zhou, Jia Wu, Jishuai Cheng, Yongzhong Duan, Wen Qian

**Affiliations:** ^1^ College of Pharmaceutics, Kunming Medical University, Kunming, China; ^2^ College of Basic Medical, Kunming Medical University, Kunming, China; ^3^ Department of Experimental Animals, Kunming Medical University, Kunming, China; ^4^ Academy of Biomedical Engineering, Kunming Medical University, Kunming, China; ^5^ Walvax Biotechnology Co., Ltd., Kunming, Yunnan, China

**Keywords:** HSV-1, antiviral, agents, immunomodulation, herpes virus

## Abstract

Herpes simplex virus type I (HSV-1) is a member of the α-herpesvirus subfamily and is capable of causing herpes simplex keratitis, herpes labialis, and herpes simplex encephalitis. HSV-1 is well known for its lytic infections at the primary sites and for establishing latency in the sensory neuronal ganglia, with occasional recurrent infections. To date, there are no approved commercially available vaccines, and anti-HSV-1 drugs such as specific or non-specific nucleotide (nucleoside) analogs and helicase-primase inhibitors have become the main clinical agents for the treatment of HSV-1 infections despite challenges from resistance. Therefore, development of new anti-HSV-1 compounds or therapies is key to addressing the issue of resistance. The present review provides an update on the progress made over approximately 60 years regarding anti-HSV-1 agents while also highlighting future perspectives for controlling HSV-1 infections.

## 1 Introduction

Herpes simplex virus type I (HSV-1) belongs to the α-herpesvirus subfamily and are is a kind of double-stranded DNA virus with an icosahedral capsid structure (Ahmad and Wilson, 2020). The infections caused by HSV are very common. Previously, reports have noted that the global HSV-1 infection rate was 64.2% in people under 50 years of age and that HSV-2 infection rate was 13.3% in people aged 15–49 years (James et al., 2020). HSV-1 commonly causes oral or ocular infections, such as herpes simplex keratitis (HSK) and herpes labialis, among others. In particular, some studies have shown that owing to the neurotropism of the herpes virus, it can also cause herpes simplex encephalitis (HSE) and Alzheimer’s disease (Rechenchoski et al., 2017; Steel and Eslick, 2015). HSV-1 can also be transmitted through oral-genital contact, and there are currently approximately 140 million reported cases in Europe, North America, and East Asia (Looker et al., 2015). During the third trimester of pregnancy, genital infections of HSV-1 can be easily passed on to newborns compared to HSV-2 (Brown et al., 1997).

The herpes virus is incurable and remains in the host for their entire life. The invasion mechanism of HSV is pretty complex, including absorption and penetration of the host cells, replication of the genetic material and protein synthesis, as well as assembly and release of the virus. Binding and entry into the cells constitutes the first step in the pathogenesis of the host infection for HSV-1, in which glycoprotein B (gB), glycoprotein D (gD), glycoprotein C (gC), and heparan sulfate (HS) proteoglycan play crucial roles. Subsequently, HSV-1 latches onto the cells using various strategies to evade the host’s innate antiviral immunity as well as some cell-survival-related pathways (Zhu and Zheng, 2020).

Because the mechanism of viral infection has been better understood, more potential therapeutic avenues have been discovered. Nowadays, HSV-1 infections are mainly treated using acyclovir (ACV) and structurally similar nucleoside analogs. However, immoderate utilization of these drugs has led to the emergence of resistant strains and undesirable effects (Birkmann and Zimmermann, 2016; Kenny et al., 2013). Therefore, researchers have focused on finding new antiviral active substances in recent years (Johnston et al., 2014; Lince et al., 2023).

## 2 Natural small-molecule substances

Natural products are considerable sources of anti-HSV active substances in recent years, and plants are the most important sources of such anti-HSV-1 active substances (Hassan et al., 2015; Treml et al., 2020; van de Sand et al., 2021; Garber et al., 2021). The structural diversity and complexities of natural products are greater than those of synthetic small molecules. Although natural products and their derivatives cannot become marketable new drugs on their own due to the difficulties associated with their synthesis and differences in extraction processes, they can provide the structural backbone for synthetic drugs. Traditional Chinese medicine (TCM) is a therapeutic strategy involving special kinds of natural products originating in China; these products have well-established theories in TCM to inform their usage as medication. Previous reviews have confirmed that such therapies and medication play important roles in the prevention and treatment of viral infections (Li et al., 2018; Li and Peng, 2013; Sun et al., 2022).

### 2.1 Phenols

Phenols having antiviral activities are present in large quantities in plant extracts. As early as 1976, it was discovered that the phenolic components of grape juice could be used to treat the polio virus (Konowalchuk and Speirs, 1976). Recently, Zannella et al. (2023) showed that the natural product Taurisolo derived from grape pomace could interact with the viral envelope; it was found that as the dose increased, the expressions of viral genes decreased, suggesting that Taurisolo may be effective in preventing and treating HSV-1 infections. Resveratrol has been demonstrated to inhibit HSV-induced activation of the nuclear factor kappa-B (NF-κB) signaling pathway as well as affect the expressions of essential immediate-early (IE), early, and late HSV genes along with the synthesis of viral DNA (Faith et al., 2006; Annunziata et al., 2018). The invasion of cells by HSV-1 leads to actin remodeling, with cofilin1 acting as a pivotal regulator of actin dynamics during this process. Pentagalloylglucose (PGG) has been shown to downregulate the expression of cofilin1 to prevent HSV-1-mediated actin remodeling (Pei et al., 2011). Psoralenic acid is a natural molecule derived from lichens and belongs to the β-orcinol depsidones; it can competitively inhibit DNA polymerase from exerting its role of inhibiting HSV-1 replication (Hassan et al., 2019). Tea is one of the most widely consumed beverages globally; it is a rich source of various substances with anti-HSV properties, including catechins extracted from green tea and epigallocatechin gallate (EGCG) that has been shown to possess anti-infective effects against HSV-1 (de Oliveira et al., 2013). Theaflavin polyphenols extracted from black tea also have anti-HSV-1 activity, especially theaflavin-3,3′-digallate, which can directly act on viral particles and inhibit binding as well as penetration of HSV-1 to the host cells (de Oliveira et al., 2015).

The structural diversity of flavonoids allows them to target the genes and proteins of viruses and cells, thereby exerting various antiviral mechanisms (Sudomova and Hassan, 2023; Hassan et al., 2022). Wang and Chen (2023) isolated mangiferin from the Chinese herb *Anemarrhena asphodeloides* and elucidated its effects on restraining the replication of HSV-1. Mangiferin at a concentration of 64 mg/L was shown to be capable of reducing the expression levels of inflammatory factors like the tumor necrosis factor (TNF)-α, interleukin (IL)-1β, and IL-6, which were remarkably increased after viral infection. Moreover, mangiferin could restore the diminished mitochondrial membrane potential caused by HSV-1 infection, thereby exerting an inhibitory effect on the virus at various stages of its lifecycle. Some researchers have confirmed that luteolin significantly inhibits the processes following viral entry into the cells rather than affecting the viral entry, assembly, or release. Luteolin has been shown to promote the oligomerization of cyclic guanosine monophosphate and adenosine monophosphate synthase (cGAS) and activate the cGAS-stimulator of the interferon gene (STING) pathway to increase the production of antiviral type I interferon (IFN-I), which exerts host innate immunity against HSV-1 infection (Wang et al., 2023a). Amentolflavone mainly inhibits the early infection of HSV-1 by not only decreasing the intracellular transport of HSV-1 from the cell membrane to the nucleus but also significantly reducing the transcription of the immediate early genes of the virus (Li et al., 2019).

Quercetin is a kind of natural flavonoid that is widely found in various plants; it can inhibit HSV infection through various mechanisms, including inhibiting viral entry as well as blocking viral binding and penetration (Hung et al., 2015). Quercetin derived from almond skin has been shown to impact the binding ability of HSV-1 to the cell membrane, block viral attachment, and inhibit viral replication (Bisignano et al., 2017). Additionally, it has been linked to selective suppression of the expression of the toll-like receptor (TLR)-3, which in turn restrains the activities of inflammatory transcription factors (NF-κB and IRF3) (Lee et al., 2017). This ultimately leads to the secretion of IFN-I and pro-inflammatory cytokines that stimulate antiviral responses within the host cells (Cho et al., 2015).

### 2.2 Alkaloids

Alkaloids with diverse structures are also important components of natural products and show inhibitory effects on a variety of viruses. Many alkaloids have been extracted from various plants, especially Chinese herbs. Berberine is an alkaloid from the Chinese herb *Coptidis rhizome*. Although it does not inhibit the invasion of cells by HSV-1, it is effective in inhibiting the syntheses of the late genes and proteins of HSV-1 (Chin et al., 2010). Five new sulfur-rich alkaloids called isatithioetherins A-E (1–5) were isolated from the root of *Isatis indigotica*. Among these, isatithioetherins 2 and 4 demonstrated antiviral activities against HSV-1 (Guo et al., 2018). *Artemisia vulgaris* L. is a traditional Chinese medicinal herb rich in phenolic acids, flavonoids, terpenoids, alkaloids, and polysaccharides. The crude extract of *A. vulgaris* L. can directly inactivate the virus or inhibit viral attachment; based on further isolation, the alkaloids naloxone and vinetine have been shown to bind more effectively to the target protein, thus exerting anti-HSV-1 activities (Xiao et al., 2023). Cepharanthine (CEP) is a naturally occurring isoquinoline alkaloid that can interfere with viral gene transcription and protein expression. Specifically, it has been shown to inhibit HSV-1 by regulating the STING/TBK1 pathway to promote autophagy rather than by interferon induction (Liu et al., 2021). Bag et al. (2014) isolated an alkaloid with the structure 7-methoxy-1-methyl-4,9-dihydro-3H-pyrido [3,4-b]indole, which is also called harmaline (HM), and demonstrated its role in combating HSV-1 by recruiting lysine-specific demethylase-1 (LSD1) and binding to the IE complex on the ICP0 promoter, ultimately leading to decreased expressions of ICP4 and ICP27. Manzamine A is a β-carbine alkaloid isolated from sponges that has been observed to target ICP0, indicating potent antiviral activity against HSV-1. Moreover, it was found that its anti-HSV-1 activity could be significantly improved by increasing its water solubility in salt form, with the unsubstituted β-carbonyl ring exhibiting greater activity (Palem et al., 2017).

### 2.3 Terpenoids

The chemical structures of terpenoids are relatively complex, and different skeleton structures represent drugs with anti-HSV activities that inhibit replication of the virus (Wimmerová et al., 2023). Triptolide (TP) is a diterpenoid triepoxide and a natural product with extensive anti-inflammatory, antitumor, and antimicrobial activities. Aliabadi et al. (2022) suggested that TP can influence HSV-1 infections by interacting with the viral transcription factors and inhibiting the synthesis of the viral mRNA, thereby limiting the production of viral proteins. The pentacyclic triterpenoid oleanolic acid has been observed to exert antiviral activity on resistant HSV-1 strains by affecting UL8, a part of the viral helicase primase complex essential for viral replication, rather than affecting viral inactivation attachment and penetration (Shan et al., 2021). The main active ingredient in *G. glabra* is glycyrrhizin, which belongs to the oleanane category of pentacyclic triterpenoid saponins; it acts as a HSV-1 polymerase inhibitor and exerts stronger antiviral effects when combined with *L. acidophilus* (Elebeedy et al., 2023).

### 2.4 Quinones

Hypericin, the main component of hypericin extracted from *Hypericum perforatum* L., can inhibit the biological activity of alkaline nuclease (AN) to inhibit HSV-1 replication (Cao et al., 2022). AN plays an important role in the replication of viral DNA and outflow of the capsid from the nucleus.

## 3 Artificial small-molecule substances

### 3.1 Non-specific broad-spectrum anti-HSV-1 nucleoside analogs

In the development of anti-HSV-1 drugs, synthetic small molecules play pivotal roles. The previously early synthesized non-specific anti-HSV-1 nucleoside analogs such as idoxuridine (5-iodo-2′-deoxyuridine (IDU or IUDR)), adenine arabinoside (9-β-D-arabinofuranosyladenine (Ara-A or vidarabine)), cytosine arabinoside (1-β-D-arabinofuranosylcytosine (Ara-C or cytarabine)), 2-deoxy-D-glucose, isoprinosine, and ribavirin (1-β-D-ribofuranosyl-1,2,4-triazole-3-carboxamid (virazole)) have been studied extensively and intensively. IDU is reported to be active against experimental herpes keratitis and has been used in thousands of clinical trials to treat HSK, of which 73% were healed over an average of 7.9 days (Maxwell, 1963); meanwhile, HSV-1 is more sensitive to IDU than HSV-2 in CEF and WI-38 cell cultures (Person et al., 1970). Ara-A was first synthesized as a potential anticancer agent in 1960 (Lee et al., 1960) and reported to be active against HSV in cell cultures in 1964 (Privât de Garilhe and Rudder, 1964). Ara-A has significant therapeutic activity against HSK in hamsters and intracerebrally inoculated HSV in mice; furthermore, it is active against central nervous system (CNS) infections in mice when administered intraperitoneally (Schabel, 1968). Ara-C is a well-known and extensively studied inhibitor of DNA synthesis that possesses significant anticancer activities in humans and animals; it has also shown broad-spectrum antiviral activity *in vitro* against DNA viruses, including HSV-1 (Buthala, 1964). Ara-C is active against herpes keratitis in rabbits, hamsters, and humans and is known to be immunosuppressive (Schabel, 1968). The anti-HSV-1 activity of 2-deoxy-D-glucose is dependent on the cell type, where it works mechanistically by altering in the ability of the virus to penetrate the cell surface (Courtney et al., 1973; Marks, 1974; Spivack et al., 1982). Ribavirin was first reported as a broad-spectrum antiviral agent in 1972 and shows anti-HSV-1 activity in KB, RK-13, CE, Vero, WI-38, and Hela cells as well as corneal epithelia from the eyes of New Zealand albino rabbits (Sidwell et al., 1972; Witkowski et al., 1972; Huffman et al., 1973, 1977).

### 3.2 Specific anti-HSV-1 nucleoside analogs

In 1977, Elion et al. (1977) reported 9-(2-hydroxyethoxymethyl) guanine (now known as ACV) as a selective antiherpetic agent and precursor molecule that, after phosphorylation by HSV-1 thymidine kinase (TK) and subsequent activation by cellular kinases, selectively inhibits viral DNA polymerase to prevent the production of infectious virions (Fyfe et al., 1978). In 1978, the non-nucleoside analog trisodium phosphonoformate (foscarnet sodium) was shown to selectively inhibit cell-free DNA polymerase activity induced by herpes viruses and have antiviral activities against HSV-1 and -2 (Helgstrand et al., 1978). Since then, several nucleoside analogs such as ganciclovir, valaciclovir, valganciclovir, panciclovir, faciclovir, and cidofovir have been developed for the treatment of HSV infections (Piret and Boivin, 2021).

The specificities and efficacies of the above anti-HSV-1 drugs are largely dependent on the TK and DNA polymerase activities of HSV-1. Once the TK or DNA polymerase gene is mutated, the virus becomes resistant to these drugs. The discovery of anti-HSV-1 agents (non-nucleoside analogs) with novel mechanisms that do not rely on TK or DNA polymerase activity is one of the main strategies for dealing with drug resistance. Nucleoside monophosphate analogs, such as cidofovir, adefovir, and brincidofovir, that are independent of TK activity have been developed (Sadowski et al., 2021). Helicase-primase inhibitors (HPIs) as new anti-HSV-1 agents inhibit the function of the HSV-1 DNA helicase-primase complex (encoded by U_L_5, U_L_8, and U_L_52) and overcome resistance to ACV in the forms of T157602, BAY 57-1293 (pritelivir), ASP2151 (amenamevir), and BILS 179 BS (Spector et al., 1998; Crute et al., 2002; Kleymann et al., 2002; Chono et al., 2010). Other enzymes involved in HSV-1 DNA synthesis, including ribonucleotide reductase (encoded by U_L_39 and U_L_40), have also been developed as targets for anti-HSV-1 agents (Lawetz and Liuzzi, 1998; Duan et al., 1998; Liuzzi et al., 1994).

### 3.3 SiRNA- and miRNA-based anti-HSV-1 agents

RNA interference (RNAi) that uses short double-stranded RNAs composed of more than 20 nucleotides to replace traditional antisense nucleic acids for post-transcriptional gene silencing regulates gene expressions to inhibit the expressions of virus-related genes, providing new pathways for gene therapy. Small interfering RNA (siRNA) can mediate sequence-specific gene silencing to achieve antiviral effects. Zhang et al. (2008) found that siRNA-1 and siRNA-4, which target VP16 and DNA polymerase, respectively, were able to effectively inhibit HSV-1 replication *in vitro*. Analogously, small hairpin RNA (shRNA) can target the U_L_28 and U_L_29 genes of the virus *in vitro* using adenovirus as the carrier to interfere with replication (Song et al., 2016).

MicroRNA (MiRNA) is an endogenous RNA that binds directly to mRNA and regulates its expression to participate in virus–host interactions (Bartel, 2009). For example, ICP4-induced miR-101 was found to decrease the expression of the RNA-binding protein G-rich sequence factor 1 (GRSF1) and weaken HSV-1 replication (Wang et al., 2016). GRSF1 is a novel target of miR-101 that promotes viral proliferation. Furthermore, it has been found that miR-101-1 as the precursor of miRNA-101 can inhibit replication *in vitro* (Sadegh Ehdaei et al., 2021). MiR-H6 also targets ICP4 to inhibit productive infection by HSV-1 and attenuate IL-6 production (Duan et al., 2012).

### 3.4 CRISPR-Cas-based anti-HSV-1 agents

Recently, *in vivo* CRISPR gene therapy was successfully performed in three patients with severe refractory disease by specifically cleaving two genes essential for the HSV-1 lifecycle, namely U_L_8 and U_L_29 (Wei et al., 2023). In a latent rabbit keratitis model, CRISPR-Cas9-mediated HSV-1 genome editing (ICP0 and ICP27) significantly eliminated viral shedding and reduced viral DNA as well as RNA expressions in trigeminal ganglia with latent infections (Amrani et al., 2024).

### 3.5 Other synthetic molecules as anti-HSV-1 agents

Some agents have the activity of inhibiting HSV-1 replication primarily by targeting certain host enzymes. Cyclin-dependent kinase 9 (CDK9) is involved in cellular gene transcription, but it can activate viral transcription. Thus, Yamamoto et al. (2014) developed a CDK9 inhibitor called FIT-039 that inhibits viral DNA replication without affecting host DNA replication. Ras-related C3 botulinum toxin substrate 1 (Rac1), which is a member of the Rho family of small GTPases, plays an important role in various cellular signaling pathways and regulates many viral infections. The molecule 6-thioguanine (6-TG) exerts potent inhibitory effects on ACV-resistant strains by targeting the recombinant Rac1 protein (Chen et al., 2021). Several enzymes of the nucleotidyl transferase superfamily (NTS) are essential for herpesvirus DNA replication, and these enzymes have recombinase and nuclease activities. Ciclopirox olamine can be dose-dependently used for the local treatment of HSV infections, possibly by interfering with the functions of one or more viral NTS enzymes (Bernier and Morrison, 2018). Bortezomib or [N-(2, 3-pyrazine) carbonyl-L-phenylalanine-L-leucine boric acid] is a dipeptide boronic acid inhibitor of the proteasome; it can act as an inhibitor in the early stages of HSV infection through two processes where it plays key roles early on, namely, transport of the HSV capsid to the nucleus and destruction of ND10 in the host cells (Schneider et al., 2019). The ND10 nuclear bodies comprise many proteins involved in normal cell growth, and their destruction is a hallmark of viral infection (Gu and Zheng, 2016). Another proteasome inhibitor MG132 inhibits HSV-1 replication by stabilizing IκB-α (negative regulator of NF-κB) to inhibit HSV-1-induced activation of NF-κB signal transduction and by overcoming the downregulation of Ras-guanine nucleotide-releasing factor 2 (Ras-GRF2) to reverse inhibition of the ERK pathway in infected cells (Ishimaru et al., 2020).

Synthetic anti-HSV-1 drugs mostly rely on nitrogen heterocycles (pyridine, pyrimidine, quinoline, etc.) and stereoscopic structures for the antiviral effects. Cetylpyridine chloride (CPC) is a quaternary ammonium compound that is widely used in hygiene products such as mouthwashes. CPC directly inactivates HSV-1, which has significant antiviral activity against the enveloped viruses but no activity against the non-enveloped viruses (Riveira-Muñoz et al., 2023), which affects the NF-κB pathway to block HSV-1 replication (Alvarez et al., 2020). Pyrithione is a zinc ionophore that has potentially multiple targets for inhibiting HSV replication, including inhibiting the expressions of the immediate early gene (ICP4) and late gene (gD) as well as intervening in the cellular ubiquitin-proteasome system (UPS) to degrade IκB-α and disrupt NF-κB activation caused by HSV-2 (Qiu et al., 2013). Wang et al. (2020b) identified that guanidine-modified BS-pyrimidine derivatives have the potential to become novel anti-HSV drugs because they can target the gB protein and cellular PI3K/Akt signaling pathways to prevent viral binding and replication. Awad et al. (2021) prepared several new cyclic and acyclic uracil nucleosides and found that these nucleosides contain a 6-substituted pyrimidine portion that enhances the biological activity, along with a free OH group that facilitates phosphorylation in viral cells; they also discovered that the activities of acyclic nucleosides were superior to those of cyclic nucleosides (Awad et al., 2021).

Amaryllidaceae alkaloid trans-dihydroalkaloid 7 was prepared by asymmetric chemical synthesis; this compound contains the key structure of C3 (3R)-secondary alcohols effectively inhibit HSV-1 infection while significantly reducing its reactivation (McNulty et al., 2016). N-[ω-(purin-6-yl) aminoalkanoyl] derivatives with anti-HSV activities were synthesized, and their chiral structures were found to be key to the antiviral activities. The inhibitory activity of the (S)-enantiomer of 7,8-difluoro-3,4-dihydro-3-methyl-2H-[1,4]benzoxazine (IC_50_ of 4.6 μM) is almost four times that of the (R)-enantiomer (IC_50_ of 18 μM) (Krasnov et al., 2019). Bernardino et al. (2012) synthesized two new 1,6-naphthidine derivatives and reported their potent anti-HSV-1 activities, with 3H-benzo[b]pyrazolo[3,4-h]-1,6-naphthyridines reducing viral production by 91% at 50 μM. Two semisynthetic cardiac glycoside derivatives were also found to have anti-HSV-1 activities without affecting the early stages of viral replication but by interfering with the later steps because they are able to completely eliminate the expressions of U_L_42 and gD proteins (Boff et al., 2020). In particular, N-docosanol does not contain nitrogenous heterocyclic structures but can still prevent the entry of viruses by directly stabilizing the cell membranes (Pope et al., 1998).

## 4 Proteins

### 4.1 Non-antibodies: cytokines and peptides

Lactoferrin is an iron-bound glycoprotein whose anti-HSV-1 activity depends on its interactions with the glycosaminoglycans on the cell surface of HS. In addition to inhibiting the adsorption of viruses, lactoferrin interferes with the spread of viruses between the cells (Berlutti et al., 2011). Antimicrobial peptides (AMPs) are widely present in the innate immune systems of organisms and have broad-spectrum antiviral properties (Memariani et al., 2020; Zannella et al., 2022). Cathelicidins is an innate host defense peptide; LL-37 is a peptide derived from human cathelicidins that has been proven to have anti-HSV activity (Lee et al., 2014). Because LL-37 containing 37 amino acids has certain limitations during synthesis, Guo et al. (2023) designed WL-1 that contains only 16 amino acids based on the structure of LL-37 to destroy the viral envelope and interfere with the viral replication cycle. RNase 7 limits HSV-1 infection of human keratinocytes by blocking viral penetration of the nucleus (Zeitvogel et al., 2024). As a wide-spectrum synthetic decapeptide, killer peptide (KP) effectively reduces the activities of ACV-resistant HSV-1 isolates by irreversibly damaging the virions before their attachment to the target cells and impeding viral adsorption (Sala et al., 2024); it has also been observed to have a synergistic effect with ACV. However, more AMPs are of animal origin. For example, Temporin-SHa (SHa) is derived from the North African ranid frog *Pelophylax saharicus* (Roy et al., 2019), and Eval418 is derived from scorpion venom polypeptide (Zeng et al., 2018).

*Aspergillipeptide* D isolated from the fungus *Aspergillus* sp. *SCSIO 41501* was found to not influence the early infection events of HSV-1 but reduced the expression level of the viral late protein gB in the viral replication phase (Wang et al., 2020b). Halovir A-E are lipophilic linear peptides extracted from marine fungi and are shown to disrupt the membrane structure of the virus to inactivate it, thus playing a role in inhibiting viral infection (Rowley et al., 2003). RLS-0071 is a 15-amino-acid anti-inflammatory peptide known as a peptide inhibitor of complement C1 (PIC1) that interferes with the complement pathway and neutrophil activation to reduce inflammation; it can also effectively reduce the mortality of HSV-1-based skin infections through synergistic effects with other antiviral drugs (Bhutta et al., 2021). The interferon (IFN) family is known to be an important component of the innate antiviral response, in which IFN-Ⅰ enhances resistance to HSV-1 infection (Borden et al., 2007). Type Ⅲ IFNs, IL-29, and IL-28A can also activate TLR-mediated antiviral pathways to reduce the expressions of viral DNA and proteins (Zhou et al., 2011).

### 4.2 Antibodies

Monoclonal antibodies (mAbs) have enormous potential in the treatment or prevention of viral infections (Backes et al., 2022). For example, dupilumab is a monoclonal antibody type Ⅰ and type Ⅱ receptor complex that targets IL-4 and IL-13 by binding to the IL-4Ra chain. Impaired viral clearance in a subgroup of atopic dermatitis patients has been shown to cause a severe HSV infection called eczema herpeticum (EH). Dupilumab helps with the treatment of atopic dermatitis by increasing INFγ and decreasing IL-4 (Traidl et al., 2023). Virus-neutralizing mAbs are one of the methods developed to target HSV infections. One mAb named 4A3 neutralizes HSV-1 at the prebinding stage by interfering with the viral binding process and effectively blocking cell-to-cell transmission of the virus (Tian et al., 2022). In a mouse model of acute retinal necrosis (ARN), the efficacy of humanized mAb hu2c targeting HSV-1/2 gB in the treatment of ocular ACV-resistant infection was studied, which could completely stop viral cell-to-cell transmission and effectively prevent ARN (Bauer et al., 2017). In addition, two recently developed humanized lgG mAbs against HSV-1/2 gB were shown to be able to bind to different epitopes of gB and induce gB internalization from the cell surface into acidic endosomes, thereby blocking viral transmission (Seyfizadeh et al., 2024).

## 5 Saccharides

Although most of the anti-HSV-1 saccharides are polysaccharides, a monosaccharide was found to have anti-HSV-1 activity. It has been reported that a rare sugar L-psicose can inhibit the adsorption of viruses at IC_50_ doses of 99.5 mM, which can be potentially used to treat HSK (Muniruzzaman et al., 2016). The structure of a polysaccharide is often complicated, and these molecules are naturally sourced from terrestrial plants, marine plants, and microorganisms. Seaweed is the most important source of polysaccharides, and many studies have shown that it has anti-HSV activity, such as the sulfuric acid polysaccharides from red algae, fucoidan from brown algae, galactan, xylan, and xylomannan (De Souza et al., 2012; Liu et al., 2020; Ray et al., 2020). Sulfated galactans like the carrageenan sulfate polysaccharide from red algae are HSV-1 inhibitors. Carrageenan has not only preventive but also virucidal effects on HSV-1, which can affect adsorption of the virus; the anti-HSV activities of different structures of carrageenan are also different. Because galactan is structurally similar to the cell surface receptor HS, it competitively binds to viruses to prevent their adsorption and penetration (Krylova et al., 2022; Davydova et al., 2023). The semirefined polysaccharides (sr-SPs) from the red algae *Rhodophyta halymenia floresii* showed strong activity with an EC_50_ of 0.68 μg/mL (Pliego-Cortes et al., 2022). Similar to galactan, xylan and mannan as well as their derivatives can inhibit HSV-1 entry into cells; the sulfated xylomannan is dominated by the α-D-mannopyranose chain, and its 2,4,6 sites are replaced with the β-D-xypyranose group to obtain HSV-1 inhibition with the EC_50_ range of 0.5–4.6 μg/mL (Ray et al., 2015; Perez Recalde et al., 2012). The same marine organism brown algae also contains a polysaccharide called fucoidan with anti-HSV-1 activity that prevents accumulation of β-amyloid in Vero cells and alleviates HSV-1-induced Alzheimer’s disease (Wozniak et al., 2015; Hadjkacem et al., 2023). Lee et al. (2004) determined 10 natural sulfated polysaccharides sourced from green algae and four synthetic sulfated xylans with anti-HSV-1 activities, and their findings suggest that certain polysaccharides can inhibit both the early and late stages of viral replication. HWE is a branched-chain isogalactose arabose polymer from the green alga *Caulerpa racemosa* that has been shown to be especially efficacious in countering ACV-resistant HSV-1 strains (Ghosh et al., 2004).

In addition to polysaccharides found in seaweed, land plants are rich sources of polysaccharides. For example, the polysaccharide from *Prunella vulgaris* is anionic in nature; it exerts inhibitory effects on HSV-1 by competing with the cell receptors and blocking TLR-mediated NF-κB activation, which in turn reduces HSV-1-induced apoptosis (Xu et al., 1999; Zhong et al., 2024). It is hypothesized that the β-glucan present in the oat crop acts as an immunomodulator, stimulating virus-infected cells to release pro-inflammatory cytokines (IL-1β, IL-6, and TNF-α) in a dose-dependent manner to reduce the risk of HSV-1 infection (Murphy et al., 2012). *Delonix regia* galactomannan (NDr) influences the early stages of infection through its virucidal effect to inhibit adsorption (de Moraes et al., 2024).

Various resin glycosides have been isolated from *Ipomoea muricata* (L.) seeds, and it was discovered that the macrolidene structure has a significant effect on cytotoxicity. The greater the number of organic acids present, the higher is the level of activity against HSV-1 (Ono et al., 2024). Echinacea polysaccharide (EP) acts as an immune stimulant to promote IFNγ production and prevent latent infection of HSV-1 (Ghaemi et al., 2009). Nuclear factor erythroid 2-related factor 2 (NRF2) plays a key role in regulating cell redox homeostasis; in response to oxidative stress, NRF2 can translocate to the nucleus, where it enhances the expressions of the antioxidant genes to resist cell death caused by viral infection. Lanatoside C (Lan C) can influence the NRF2 pathway to inhibit HSV-1 infection. Nevertheless, it does not reduce the expression level of NRF2; instead, it plays an antiviral role by promoting nuclear translocation of NRF2 and activating the downstream protein-coding genes, including *HO-1* and *NQO-1* (Wu et al., 2024).

Microorganisms are also reservoirs of polysaccharides, as exemplified by the K5 polysaccharide derivative of *Escherichia coli*. This is composed primarily of glucose and exhibits structural similarities to heparin, which can destroy HSV-1 particles and impede early viral adsorption onto the host cells (Pinna et al., 2008). Cyanobacterium exopolysaccharides (EPS) have been established as a means of inhibiting the adsorption and replication of viruses. EPS hinders viral activity through ionic interactions by utilizing the positively charged glycoproteins and negatively charged sulfated EPS on the surface of the virus (Saad et al., 2024).

In addition to naturally extracted polysaccharides, artificially synthesized and modified natural polysaccharides have good antiviral effects. Wouk et al. (2022) showed that the sulfonated (1→6)-β-d-glucan lasiodiplodan has antiviral properties against ACV-resistant strains and can inhibit DNA and protein syntheses. Sulfonated and carboxymethylated β-glucan derivatives can inhibit HSV-1; through comparison of the antiviral activity with that of sulfonated botryosphaerans, it was found that the carboxymethyl component plays a crucial role in antiviral activity (Lopes et al., 2021).

There are many synthetic polysaccharide substances that can affect the binding of viruses to cellular receptors, such as the series of cationic dextran derivatives (EX_x_DS_y_) synthesized by researchers at the University of Vagellone. Pachota et al. (2017) and Gangji et al. (2018) screened a series of compounds called non-saccharide glycosaminoglycan mimics (NSGMs) by targeting HSV-1 to affect gD to inhibit the binding and entry of viruses to the host cells. Heparin has broad-spectrum antiviral effects because it non-specifically blocks viral surface proteins. Heparin modified by magnesium chloride has more potent anti-HSV-1 effects than ACV, which can further reduce viral replication (Mese et al., 2021). After infection, the virus can promote the expression of human heparanase (Hpse) to remove HS and promote the spread of the virus. Therefore, Chopra et al. (2023) synthesized a series of HS-oligosaccharides as Hpse inhibitors and found that the hexasaccharides and octasaccharides play potent roles in inhibiting this enzyme.

## 6 Lipid substances

One study explored the structure–activity relationships of polyhydroxylated sulfated steroids extracted from marine echinoderms and their effects on anti-HSV-1 activity (Pujol et al., 2016). One of the steroids (2β,3α-dihydroxy-6e-hydroxy-5α-cholestan-2,3-disodium disulfate) has good broad-spectrum antiviral activity (HSV-1, HSV-2, and pseudorabies virus strains, including ACV-resistant variants), and its mechanism is not likely to block the initial steps of the virus but act on gD to influence the subsequent events in the course of infection. The sulfate groups at C-2(β) and C-3(α) as well as the substitution of hydroxyl, sulfate, keto, or oxime groups at the C-6 position are important for the antiviral activities of synthetic cholestanes. The signal transducer and activator of transcription-3 (STAT3) regulates viral reactivation and is influenced by the upstream negative regulator suppressor of cytokine signaling 1(SOCS1). Wedelolactone (WDL) can directly destroy the viral envelope structure and inactivate viral particles on HSV-1; it also exerts an immune role by acting on the TBK1/IRF3 and SOCS1/STAT3 pathways to reduce HSV-1 infection and inflammatory responses (Wang et al., 2023b). Shamsian et al. (2024) found that the *Cassiopea andromeda* jellyfish tentacle extract (TE) inhibited HSV-1 infection where more than half of the active compounds contained steroid structures, and molecular docking studies of this extract revealed that oxosteroid binds better to thymidine kinase.

## 7 Other activities

In addition to the agents mentioned above, there are some substances that are not classified clearly, which could be combinations of two substances or new types of materials or even just crude extracts. Mitochondrial release of reactive oxygen species (ROS) can activate the nod-like receptor protein 3 (NLRP3) inflammasome, which then activates the downstream pro-inflammatory cytokines, such as IL-1β, to enhance the host innate immune responses. The main mechanism by which Korean chestnut honey (KCH) inhibits HSV-1 infection is by ameliorating the mitochondrial dysfunction caused by HSV-1 infection, regulating the ROS-NLRP3 inflammasome pathway, and destroying the viral particles before interacting with the cells to play a virus-killing role (Kwon et al., 2023). Pennisi et al. (2023) used n-hexane to extract Californian natural raw (NRRE) and roasted unsalted (RURE) pistachios and found that their extracts were effective against HSV-1 as they prevented the virus from binding to the cell, thereby influencing viral DNA synthesis and accumulation of the ICP0, U_L_42, and U_S_11 viral proteins.

Silver nanoparticles (AgNPs) have broad-spectrum antiviral activity and play significant roles in multiple stages of the viral lifecycle. They can act directly on the HSV-1 particles to disrupt their envelope structures, downregulate the expressions of the IE, early, and late genes, interfere with mRNA transcription and protein expression, and reduce the inflammatory cytokines caused by HSV-1 (Pan et al., 2022). Thermally expanded graphite and CuO nanocomposite (10 μg/mL) has been shown to be a promising candidate for the treatment of HSV-1 infection as it can inhibit 99% of the virus production (Hamidzade et al., 2024). Furthermore, the nanoparticles can be modified with other agents, such as EGCG-modified AgNPs to improve the contact area for EGCG with HSV-1, thus exerting a greater anti-HSV-1 effect (Krzyzowska et al., 2023). Selenium nanoparticles synthesized from the aqueous extract of the brown alga *Polycladia myrica* as an anticancer agent was also shown to have a virucidal effect, inhibiting 35.25% of HSV-1 in Vero cells (Abo-Neima et al., 2023).

## 8 Conclusion and future prospects

**FIGURE 1:**
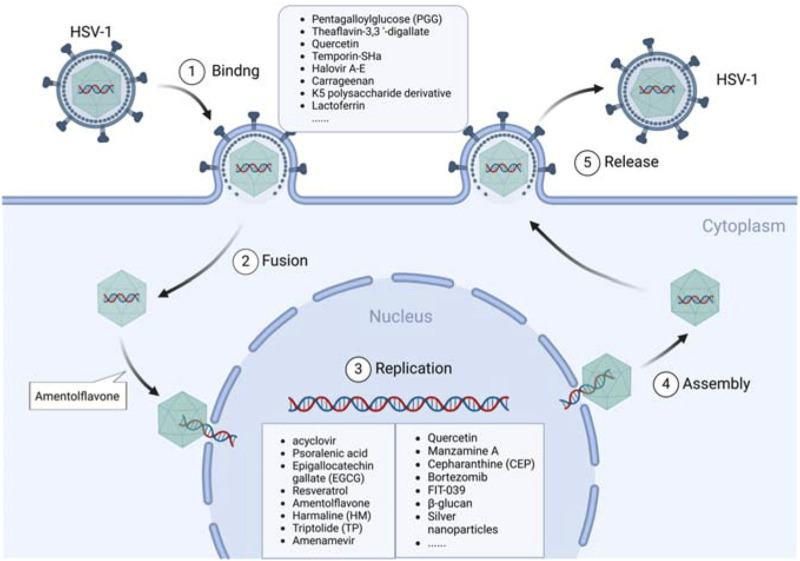
Anti-HSV-1 agents exert their antiviral effects by targeting the viral lifecycle. HSV-1 infects host cells through a chain of roughly five processes: binding, fusion, replication, assembly, and release. Most of the active substances play inhibitory roles during the cell binding and viral DNA replication stages.

**FIGURE 2:**
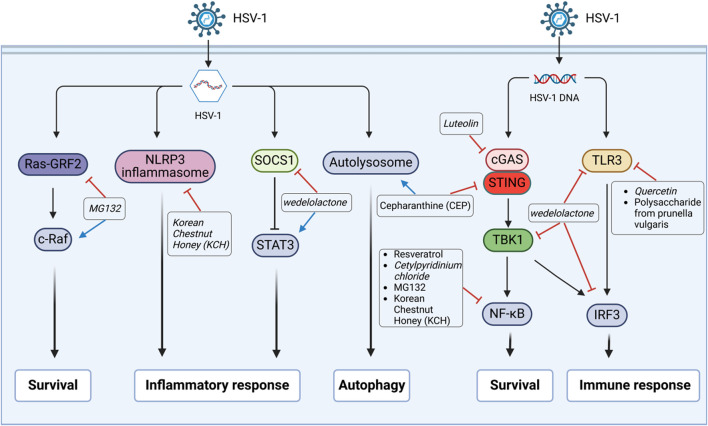
Anti-HSV-1 agents exert their antiviral effects by regulating the host signaling pathways. Following HSV-1 infection, the active substances exert inhibitory effects through various signaling pathways. The HSV-1 DNA uses the toll-like receptor-3 (TLR3) to stimulate cells to produce the transcription factors interferon regulatory factor 3 (IRF3) or nuclear factor kappa-B (NF-κB), which then induce expressions of the type I interferon and pro-inflammatory cytokine genes. Cyclic GMP-AMP synthase (cGAS) can sense the damaged DNA and activate the stimulator of interferon gene (STING), which then activates TANK-binding kinase 1 (TBK1) and eventually activates IRF3 or NF-κB to play autoimmune roles. It can mediate pyrodeath and IL-1β secretion by influencing the formation of NLRP3 inflammasomes. Signal transducer and activator of transcription 3 (STAT3), which is inhibited by the upstream suppressor of cytokine signaling 1 (SOCS1), can regulate cellular activities like cell growth and immune responses. Ras-protein-specific guanine nucleotide-releasing factor 2 (Ras-GRF2) activates c-Raf to regulate the signaling of extracellular regulated protein kinases (ERK1/2), while ERK inhibition promotes viral replication.

Based on their action mechanisms, anti-HSV-1 agents can be divided into three main classes: direct antiviral effect (direct inactivation or inhibition at various stages of the viral lifecycle, such as adsorption, binding, penetration, replication, and release) as shown in Figure 1; indirect antiviral effect by regulating the physiological processes of host cells, such as immune responses, inflammasome responses, survival, and autophagy, as shown in Figure 2; combination of direct and indirect effects. Herein, we have summarized the reported some anti-HSV-1 agents, and their details are shown in Supplementary Table S1. According to the available data, the sulfated polysaccharides (SP2, SP5, and SP9) from green algae have very strong anti-HSV-1 activities in Vero cells, with SI values >10,000; in contrast, the anti-HSV-1 SI of ACV is 1,200 (Lee et al., 2004). Further validations are needed in animal models and through clinical trials. At the same time, the targets of the sulfated polysaccharides need to be identified.

Anti-HSV-1 agents have been developed for decades, from broad-spectrum to specific agents and from a single type of molecule to a combination of substances, and each achievement has brought great hope to people. However, drug resistance remains a significant concern, reminding us that the development of novel anti-HSV-1 agents is necessary. There appears to be no single approach to solving the problem of viral drug resistance, and the development of individual or combination of drugs with different antiviral mechanisms appears to be feasible. For example, DB111 as a CRISPR-Cas-system-based drug for the treatment of HSK has recently been tested in clinical trials (NCT06474416 and NCT06474442). It is worth noting that drug resistance research should be conducted throughout the drug development process. Prodrugs at the basic research stage lack in-depth investigations on the action mechanisms, which can make drug resistance studies difficult. These characteristics of prodrugs determine that safety and efficacy should be the focus of attention during the basic research stage.

Similar to other drugs, anti-HSV-1 drugs involve large investments and lengthy development cycles, which is why we can find many anti-HSV-1 substances in basic research, although very few of these have progressed to the clinical stage of research. It would be a worthwhile effort to find drugs with anti-HSV-1 activities among the existing drugs in the market. At present, such studies have been reported in literature (Bautista et al., 2024), which is of great significance for accelerating the development and commercialization of anti-HSV-1 drugs. HSV-1 has developed clever mechanisms to resist certain antiviral drugs through latency, mutation, and so on. The research hope here is that anti-HSV-1 drugs can also be smart to work against different kinds of mutant viruses. After all, the humans who develop and control such antiviral tools are innately smarter than the viruses.
