# Supplementary material for: Anti-HSV-1 agents: an update
Source: Front Pharmacol. 2025 Jan 21;15:1451083. doi: 10.3389/fphar.2024.1451083 (PMC11808302; doi:10.3389/fphar.2024.1451083)
Supplement: Supplementary file 1 [file Table1.docx]

Table 1 The summary of anti-HSV-1 agents

| Substance name | Experimental details | | | | Action mechanism | Reference |
| --- | --- | --- | --- | --- | --- | --- |
|  | Models | Strains | Effect | SI |  |  |
| Phenols | | | | | | |
| Taurisolo | Vero cells | HSV-1 SC16 | IC_50_ = 0.097 µg/mL | No mention | 1.Interaction with viral envelope  2.Inhibition of viral gene expression | (Zannella et al., 2023) |
| Resveratrol | Vero cells | acyclovir resistant HSV-1 (ACV-R HSV-1) | 219 μM resveratrol reduced HSV titers by 81.5%. | No mention | 1.Inhibition of HSV-induced NF-κB activation  2.Influence on the gene expression necessary for the synthesis of viral DNA | (Faith et al., 2006;Annunziata et al., 2018) |
| Pentagalloylglucose (PGG) | Vero and MRC-5 cells | HSV-1 F | EC_50_ = 4.12 ± 0.67 μM (PGG), EC_50_ = 0.98 ± 0.24 μM (ACV); CC_50_ = 745.02 ± 4.51 μM (PGG), CC_50_ = 2747.52 ± 77.82 μM (ACV) | SI = 180.83 (PGG), SI = 2803.59 (ACV) | 1.Inhibition of entry by down-regulating cofilin1  2.Inhibition of replication and transcription of DNA and mRNA | (Pei et al., 2011) |
| Psoralenic acid (PA) | Vero cells | HSV-1 KOS | IC_50_ = 1.9 µM (PA), IC_50_ = 2.6 µM (ACV) | SI = 163.2 (PA), SI = 119.2 (ACV) | Competitive inhibition of DNA polymerase | (Hassan et al., 2019) |
| Epigallocatechin gallate (EGCG) | Vero cells | HSV-1 with GFP flag | 50 µM p-EGCG caused a >99% decrease in HSV-1 PFU | No mention | Inhibition of ICP0 expression | (de Oliveira et al., 2013) |
| Theaflavin-3,3 '-digallate (TF3) | Vero and A549 cells | HSV-1 with GFP flag | EC_50_ = 20 μM | SI = 5.625 | Acting directly on virus particles and inhibiting binding | (de Oliveira et al., 2015) |
| Flavonoid | | | | | | |
| Mangiferin | Female BALB/c mice | HSV-1 | The cumulative mortality of mice was 40.0% and the relative protection rate was 55.5% (HSV-1 and 4 g/kg mangiferin). | No mention | 1.Reduction in the expression of inflammatory cytokines TNF-α, IL-1β and IL-6  2.Restoration of mitochondrial membrane potential levels | (Wang and Chen, 2023) |
| Luteolin | Vero, HaCaT, BV2, RAW264.7 and SH-SY5Y cells | HSV-1 F and ACV-resistant strains | CC_50_ of cells was 347.93 ± 18.78 μM, 41.66 ± 0.33 μM, 46.89 ± 0.02 μM, 45.98 ± 1.00 μM and 32.35 ± 0.97 μM, respectly | No mention | Promotion of cGAS-STING pathway to increase production of antiviral type I interferon | (Wang et al., 2023) |
| Amentolflavone (AF) | Vero and SK-N-SH cells | HSV-1, HSV-1/Blue, HSV-1/106 and HSV-1/153 | CC_50_ >100 μM (AF and ACV); EC_50_ = 22.13 ± 0.38 μM (HSV-1), EC_50_ = 11.11 ± 0.71 μM (HSV-1/106), EC_50_ = 28.22 ± 2.51 μM (HSV-1/153), EC_50_ = 25.71 ± 3.97 μM (HSV-1/Blue) | SI >4.52 (HSV-1), 9.00 (HSV-1/106), 3.54 (HSV-1/153), 3.89 (HSV-1/Blue) | 1. Reduction of intracellular transport of HSV-1 from cell membrane to nucleus,  2. Inhibition of the transcription of direct early genes of the virus | (Li et al., 2019) |
| Quercetin | Vero and Raw264.7 cells | HSV-1 | Decrease in plaque formation in Vero cells when they were incubated with infected cell lysates treated with quercetin (50% decrease for 10 μg/ml quercetin, 80% decrease for 20 μg/ ml quercetin, and 90% decrease for 30 μg/ml of quercetin) | No mention | 1. Inhibition of virus entry, binding, and penetration,  2. Inhibition of the expression of TLR-3 and induction of the secretion of type I interferon and proinflammatory cytokines | (Cho et al., 2015;Hung et al., 2015;Bisignano et al., 2017;Lee et al., 2017) |
| Alkaloids | | | | | | |
| Berberine | Vero cells | HSV-1 F | CC_50_ = 13.2 ± 1.6 mg/mL (Berberine), CC_50_ = 7.8 ± 0.9 ×10-2 mg/mL (ACV); EC_50_ = 8.2 ± 1.2 ×10-2 mg/mL (Berberine), EC_50_ = 2.2 ± 0.5 ×10-4 mg/mL (ACV) | SI = 160.97 (Berberine), SI = 354.54 (ACV) | Inhibition of late gene expression | (Chin et al., 2010) |
| Naloxone and vinvinetine | Vero and MA-104 cells | HSV-1 F, HSV-1/Blue and HSV-1/153 | EC_50_ = 85.6 μg/mL (Vero), EC_50_ = 62.9 μg/mL (MA-104) | No mention | 1.Inactivation of the virus  2.Targeting the gB protein and inhibition of attachment | (Xiao et al., 2023) |
| Cepharanthine (CEP) | Vero and HeLa cells | HSV-1 | CC_50_ = 5.4 μg/mL (Vero), CC_50_ = 9 μg/mL (HeLa); EC_50_ = 0.835 μg/mL | SI = 6.5 (Vero), SI = 10.8 (HeLa) | 1.Inhibition of viral gene transcription and protein expression  2. regulation of the STING/TBK1/P62 pathway to promote autophagy | (Liu et al., 2021) |
| Harmaline (HM) | Vero cells | HSV-1 F | CC_50_ = 30 ± 0.32 μg/mL (HM); EC_50_ = 1.1 ± 0.1 μg/mL (HM), EC_50_ = 2.1 ± 0.21 μg/mL (ACV) | SI = 27.27 (HM) | 1.Interference with the recruiting lysine-specific demethylase-1 (LSD1) to inhibit replication  2.Inhibition of early gene expression | (Bag et al., 2014) |
| Manzamine A | SIRC | HSV-1 | EC_50_ = 5.6 µM; MIC = 1 µM | No mention | Inhibition of replication by targeting ICP0 | (Palem et al., 2017) |
| Terpene | | | | | | |
| Triptolide | PRK, A549 and Vero cells | HSV-1 | CC_50_ = 4.454 μM (A549), CC_50_ = 21.46 μM (PRK); EC_50_ = 0.05 μM (A549), EC_50_ = 0.065 μM (PRK) | SI = 89.08 (A549), SI = 330.15 (PRK) | Inhibition of viral transcription | (Aliabadi et al., 2022) |
| Oleanolic acid | Vero, SH-SY5Y and HaCaT cells | HSV-1 F, HSV-1/106, HSV-1/Blue and HSV-1/153 | EC_50_ of Oleanolic acid were 39.05 ± 0.561 μM (Vero), 20.5 ± 0.325 μM (SH-SY5Y) and 37.06 ± 0.401 μM (HaCaT), ACV EC_50_ >50 μM (Vero, SH-SY5Y and HaCaT); EC_50_ of Oleanolic acid were 4.712 ± 0.321 μM (HSV-1 F), 12.89 ± 0.681 μM (HSV-1/106), 12.89 ± 0.681 μM (HSV-1/153) and 13.09 ± 0.642 μM (HSV-1/Blue), EC_50_ of ACV were <0.5625 μM (HSV-1 F) and >20 μM (HSV-1/106, HSV-1/Blue and HSV-1/153) | No mention | Inhibition of replication by affecting UL8 | (Shan et al., 2021) |
| glycyrrhizin | Vero cells | HSV-1 | HSV-1 log reduction in titer of 0.25 (G. glabra), 0.5 (L. acidophilus suspension), 1.25 (L. acidophilus supernatant) and 1.25 (Combination of L. acidophilus and G. glabra) | No mention | HSV-1 polymerase inhibitor | (Elebeedy et al., 2023) |
| Quinones | | | | | | |
| Hypericin | Vero cells | HSV-1 F and HSV-1 SM44 (an HSV-1 strain of a clinical isolate) | EC_50_ = 2.59 ± 0.08 μM (HSV-1 F), and EC_50_ = 2.94 ± 0.10 μM (HSV-1 SM44); CC_50_ = 34.06 ± 2.87 μM | SI = 13.5 (HSV-1 F), and SI = 11.58 (HSV-1 SM44) | Inhibition of the biological activity of alkaline nuclease and HSV-1 replication | (Cao et al., 2022) |
| Artificial small molecule substance | | | | | | |
| 9-f3-D-arabinofurano  syladenine (ara-A) | Chick embryo fibroblasts (CEF), WI-38 and HeLa cells | HSV WI-38 | Zone of inhibition were 26 ± 3.3 mm (CEF), 24 ± 4.0 mm (WI-38) and 20 ± 1.4 mm (HeLa) | No mention | DNA synthesis inhibitor | (Schabel, 1968;Person et al., 1970) |
| Ara-C | Rabbit kidney cells | HSV-1 | Data was not shown. | No mention | DNA synthesis inhibitor | (Buthala, 1964) |
| 2-deoxy-D-glucose | Primary rabbit kidney (PRK) cells, WI-38 and Rat brain cells (RB) | HSV-1 | Minimal inhibitory concentration (MIC) was 40 μg/mL (PRK), 40 μg/mL (WI-38) and 20.7 μg/mL (RB) | No mention | Reduction of the ability of viruses to penetrate the cell surface | (Marks, 1974) |
| Ribavirin | Vero cells | HSV-1 HF | Concentrations of ribavirin as low as 3.2 pg/mL resulted in virus titer reductions of over 1 log, while markedly inhibiting the development of viral cytopathic effects (CPE) in the cells | No mention | No mention | (Huffman et al., 1977) |
| Acyclovir (ACV) | Vero and HeLa S-3 cells | HSV-1 KOS | EC_50_ = 0.1 μM | No mention | DNA polymerase inhibitor | (Elion et al., 1977) |
| Phosphonoformic acid (PFA) | Rabbit kidney cells | HSV-1 8/11, HSV-1 KJ502, HSV-1 90155, HSV-1 V8523 and HSV-1 V8663 | Percentage of inhibition of 100 μM PFA = 95 (HSV-1 8/11), 92 (HSV-1 KJ502), 95 (HSV-1 90155); Percentage of inhibition of 500 μM PFA >99.99 (HSV-1 V8523 and HSV-1 V8663) | No mention | No mention | (Helgstrand et al., 1978) |
| T157602 (2-amino thiazole) | Vero, human foreskin fibroblasts (HFF cells) and Jurkat cells | HSV-1 with mutated UL5, UL8, and UL52 genes | HSV helicase activity EC_50_ = 5 μM, primase activity EC_50_ = 20 μM | No mention | Interactions with the helicase component of the UL5-UL8-UL52 Complex | (Spector et al., 1998) |
| BILS 179 BS | BHK-21/C13 cells | HSV-1 | EC_50_ = 0.027 μM; CC_50_ = 59 μM | SI = 2200 | Inhibition of replication | (Crute et al., 2002) |
| BAY 57-1293 (Pritelivir) | Vero cells | HSV-1 F | EC_50_ = 0.02 μM (BAY 57-1293), EC_50_ = 1 μM (ACV) | SI = 2500 (BAY 57-1293), SI = 25 (ACV) | Inhibition of replication | (Kleymann et al., 2002) |
| ASP2151 (Amenamevir) | Human embryonic fibroblast (HEF) cells | HSV-1 KOS | EC_50_ = 0.036 ± 0.0047 μM; CC_50_ > 30 μM | SI >638 | Inhibition of replication by affecting UL5 and UL52 | (Chono et al., 2010) |
| BILD 1351 SE | Vero and baby hamster kidney (BHK) | HSV-1 F HA14 | EC_50_ of BILD 1351 SE in serum-starved and nonserum-starved cells were 2 ± 0.9 and 4.1 ± 1.6 μM, respectively. EC_50_ of ACV was 2.7 ± 0.9 μM | No mention | Ribonucleotide reductase inhibitor | (Lawetz and Liuzzi, 1998) |
| BILD 1633 SE | Vero cells | HSV-1 F, HSV-1 KOS, and two ACV-resistant strains, dlsptk and PAAr5 | EC_50_ of BILD 1633 SE weer 0.35 ± 0.10 μM (F), 0.43 ± 0.06 μM (KOS), 0.46 ± 0.09 μM (dlsptk) and 0.14 ± 0.02 μM (PAAr5). EC_50_ of ACV were 2.7 ± 1.0 μM (F), 5.23 ± 1.0 μM (KOS), 60.2 ± 12.9 μM (dlsptk) and 17.2 ± 2.9 μM (PAAr5). BILD 1633 SE CC_5_0 = 14 μM | SI = 40 (F), 32.56 (KOS), 30.43 (dlsptk) and 100 (PAAr5) | Ribonucleotide reductase inhibitor | (Duan et al., 1998) |
| BILD 1263 | BHK | HSV-1 F | EC_50_ = 3 μM, CC_50_ = 100 μM | SI = 33.33 | Ribonucleotide reductase inhibitor | (Liuzzi et al., 1994) |
| siRNA-1 and siRNA-4 | Vero cells | HSV-1 | cytopathic effect (CPE) of 90% (siRNA-1) and >70% (siRNA-4) | No mention | Inhibition of replication | (Zhang et al., 2008) |
| Adenovirus-mediated shRNA | Vero cells | HSV-1 F | Data was not shown. | No mention | Inhibition of HSV-1 replication | (Song et al., 2016) |
| MiR-101-2 | HeLa cells | HSV-1 | Data was not shown. | No mention | Reduction of the expression of GRSF1 and attenuation of HSV-1 replication | (Wang et al., 2016) |
| MiR-101-1 | HeLa cells | HSV-1 | Data was not shown. | No mention | Inhibition of HSV-1 replication | (Sadegh Ehdaei et al., 2021) |
| MiR-H6-3p | HCECs | HSV-1 F | Data was not shown. | No mention | Attenuation of IL-6 production and inhibition of HSV-1 replication | (Duan et al., 2012) |
| AAV8(Y733F)-SaCas9 or AAV9-SaCas9 vector | HEK293FT and Vero cells | HSV-1 | Data was not shown. | No mention | Editing the HSV-1 gene to reduce infection | (Amrani et al., 2024) |
| FIT-039 | Vero cells | HSV-1 F | EC_50_ = 0.69 μM | No mention | Inhibition of HSV-1 replication | (Yamamoto et al., 2014) |
| 6-thioguanine (6-TG) | Human corneal epithelial cells (HCECs) | HSV-1 HF | EC_50_ = 0.104 ± 0.013 μM (6-TG), EC_50_ = 1.253 ± 0.073 μM (ACV); CC_50_ = 700.06 ± 16.3 μM (6-TG), CC_50_ >5000 μM (ACV) | SI = 6,796.69 | Inhibition of HSV-1 replication by targeting Rac1 protein | (Chen et al., 2021) |
| Ciclopirox olamine | BALB/c mice | HSV-1 KOS and 17 | the higher doses of ACV and ciclopirox controlled virus replication in the corneal epithelium better than application of saline. | No mention | Interference with the function of viral NTS enzymes | (Bernier and Morrison, 2018) |
| Bortezomib | Vero cells | HSV-1 KOS, HSV-1/ACGr5, HSV-1/PAAr5, HSV-1/dlsptk, HSV-1 H129 | EC_50_ = 21.3 ± 4.10 nM (HSV-1 KOS), EC_50_ = 10.1 ± 3.82 nM (ACGr5), EC_50_ = 3.70 ± 0.98 nM (AAr5), EC_50_ = 25.1 ± 8.42 nM (dlPstTK-), EC_50_ = 50.6 ± 12.71 nM (H129) | No mention | Inhibition of HSV-1 penetration and replication | (Schneider et al., 2019) |
| MG132 | Vero cells | HSV-1 HF | Decreased by approximately 35% at a higher concentration (0.75 μM) of MG132 treated cells compared with vehicle treated cells | No mention | Inhibition of HSV-1 replication via NF-κB pathway and overcoming down-regulation of Ras-GRF2 to reverse ERK signaling pathway inhibition | (Ishimaru et al., 2020) |
| Cetylpyridine chloride (CPC) | Vero E6 and 293TT cells | HSV-1 | CPC (mean viability = 71.9% ± 17.2; p-value = 0.0742); PerioAid (mean viability = 83.3% ± 4.1; p-value = 0.059) | No mention | 1.Inactivation of HSV-1 particle  2.Influence on the Nf-κB pathway | (Alvarez et al., 2020;Riveira-Muñoz et al., 2023) |
| Zinc ionophores pyrithione | HEC-293T, Vero, Hela and HEC-1-A cells | HSV-1 HF | Data was not shown. | No mention | Inhibition of replication by affecting the expression of ICP4 and gD | (Qiu et al., 2013) |
| Guanidine-modified BS-pyrimidine derivatives | Vero E6, Hep-2 and HeLa cells | HSV-1 F | CC_50_ = 119.9 ± 2.7 μM (derivative), CC_50_ = 459.5 ± 17.3 μM (ACV); EC_50_ = 1.8 ± 0.1 μM (derivative), EC_50_ = 9.6 ± 0.8 μM (ACV) | SI = 66.6 (derivative), SI = 47.9 (ACV) | Prevention of viral binding and replication by targeting gB and cellular PI3K/Akt signaling pathways | (Wang et al., 2020a) |
| Novel cyclic and acyclic uracil nucleosides | Vero cells | HSV-1 KOS | EC_50_ = 15.19 (compounds 8), 15.76 (Cpd. 6), 25.23 (Cpd.4), 13.96 (ACV); CPE Inhibition (%) with 18 µg/mL = 22.35 ± 2.25 (Cpd.4), 41.25 ± 4.05 (Cpd.8), 45.90 ± 3.40 (Cpd.6), 49.80 ± 3.40 (ACV). | No mention | No mention | (Awad et al., 2021) |
| Amaryllidaceae alkaloid trans-dihydroalkaloid | Vero cells and iPSC-neurons | HSV-1 | EC_50_ = 0.10 μM (concentration of 7); EC_50_ = 0.07 μM (ACV) | No mention | Reduction of the reactivation of HSV-1 | (McNulty et al., 2016) |
| (S) -enantiomer of 7,8-difluoro-3,4-dihydro-3-methyl-2H-[1,4]benzoxazine (S-10e) | Vero cells | HSV-1 and acyclovir-resistant HSV-1/L2/R | CC_50_ = 293.48 ± 15.55 μM (S-10e), CC_50_ >444 (ACV); EC_50_ = 4.6 μM (S-10e), EC_50_ >444 (ACV) | SI = 64 (S-10e), SI = 1 (ACV) | No mention | (Krasnov et al., 2019) |
| 3H-benzo[b]pyrazolo[3,4-h]-1,6-naphthyridines (compound 1h ) | Vero cells | HSV-1 | Reducing viral production by 91% at 50μM; EC_50_ = 0.07 μM (1h), EC_50_ = 1.09 ± 0.25 μM (1h); CC_50_ = 600 μM (1h), CC_50_ = 960 ± 156 μM (ACV) | SI = 8,571 (1h), SI = 880 (ACV) | No mention | (Bernardino et al., 2012) |
| Semi-synthetic cardiac glycoside derivatives (C10 and C11) | Vero cells | HSV-1 KOS and 29-R | EC_50_ of 0.23 (C10) and 0.24 µM (C11) against KOS strain; 0.18 (C10) and 0.19 µM (C11) against 29-R strain | SI for C10 and C11 were as follows: 1,304 and 1,250 (KOS); 1,667 and 1,579 (29-R); acyclovir (KOS) 449 | Elimination of the expressions of UL42 and gD | (Boff et al., 2020) |
| Protein | | | | | | |
| Lactoferrin (Lf) | Vero cells | HSV-1 | Intracellular fluorescent-labeled spots 90 min post-infection of Control and Lf were 7.1%, 36.7% in cell periphery, 44.8%, 62.3% in cytoplasm and 47.6%, 1.0% in nucleus, respectively | No mention | 1.Inhibition of the adsorption  2.Interference with the spread of viruses between cells | (Berlutti et al., 2011) |
| LL-37 | HCEs | HSV-1 F | LL 37 Viral Titer of 81.60 (10 μg/ml) and 510.00 (20 μg/ml) at 24 hpi.; 5.17 (10 μg/ml) and 136.36 (20 μg/ml) at 48 hpi.; entry blocker (EB) Viral Titer of 3.64 (10 μg/ml) and 6.38 (20 μg/ml) at 24 hpi.; 1.15 (10 μg/ml) and 2.68 (20 μg/ml) at 48 hpi | No mention | Inhibition of binding | (Lee et al., 2014) |
| WL-1 | C57 female mice, U251 cells and Vero cells | HSV-1 | EC_50_ = 10 μM (WL-1); the survival rate of HSV-1-infected mice in the presence of WL-1 was ~90% by day 20, which was markedly higher than that of mice without WL-1 administration | No mention | No mention | (Guo et al., 2023) |
| RNase 7 | Baby hamster kidney (BHK)‐21 and Vero cells | HSV-1 17^+^ and HSV-1-GFP | RNase 7 reduced HSV1-GFP expression by 80% at 1.35 µM | No mention | Blockade of HSV-1 penetration into the nucleus | (Zeitvogel et al., 2024) |
| Killer Peptide (KP) | Vero cells | HSV-1 and the ACV-resistant HSV-1 isolates | CC_50_ = 401.6 µg/mL (KP), CC_50_ = 862.5 µg/mL (ACV); EC_50_ = 13.6 µg/mL (KP), EC_50_ = 0.5 µg/mL (ACV) | SI = 29.6 (KP), SI = 1742.5 (ACV) | Direct killing the virus and inhibition of adsorption | (Sala et al., 2024) |
| Temporin-SHa | Vero cells | HSV-1 | Temporin-SHa was cytotoxic at 20 µM, showing a 77% cell viability, while Tb significantly decreased cell viability from 60 µM (65% cell viability) compared to untreated cells | No mention | Acting directly on virus particles and inhibit binding | (Roy et al., 2019) |
| Eval418 | Vero cells | HSV-1 F | CC_50_ = 68.50 μg/mL (Eval418), CC_50_ = 106.68 μg/mL (Eval418-FH5); EC_50_ = 2.48 μg/mL (Eval418), EC_50_ = 0.86 μg/mL (Eval418-FH5) | SI = 27.62 (Eval418), SI = 124.05 (Eval418-FH5) | Viral inactivation activity and enhanced attachment inhibitory activity | (Zeng et al., 2018) |
| Aspergillipeptide D | Vero cells | HSV-1 F, HSV-1/Blue, HSV-1/106 and HSV-1/153 | CC_50_ = 208.723 ± 9.717 μM; HSV-1 F EC_50_ = 7.928 ± 0.511 μM (Aspergillipeptide D), EC_50_ = 3.606 ± 0.302 μM (ACV); The EC_50_ of Aspergillipeptide D against HSV-1/ 153, HSV-1/106, and HSV-1/Blue were 8.277 ± 1.249 μM, 10.486 ± 0.929 μM, and 7.9875 ± 0.616 μM, respectively | SI = 26.327 | Inhibition of the expression of late protein gB | (Wang et al., 2020b) |
| Halovir A-E | Vero cells | HSV-1 | EC_50_ = 10 ~ 20 μg/mL | No mention | Destruction of the membrane structure of the virus and inactivation of the virus | (Rowley et al., 2003) |
| RLS-0071 | Female BALB/cJ mice | GFP-HSV-1  17^+^, Acyclovir-resistant GFP-HSV-1 | Vehicle DMSO-treated animals exhibited a 0% survival rate within  14 days; RLS-0071-treated animals showed a 53.3% rate of survivability p.i. compared to the vehicle-treated control animals across 14 days | No mention | (Inhibitor of complement C1)  Acting on inflammatory pathways and plays a role in innate immunity | (Bhutta et al., 2021) |
| IL-29 and IL-28A | CHP212 cells and NT2-N neurons | HSV-1 17^+^ | Data was not shown. | No mention | TLR-mediated antiviral pathway | (Zhou et al., 2011) |
| Dupilumab | Patients aged 18 or older with HSV IgG positivity | HSV-1 | Data was not shown. | No mention | Playing a role in innate immunity by targeting IL-4 and IL-13 | (Traidl et al., 2023) |
| 4A3 | U2OS, Vero, 293T, HEL299, HFF-1, HeLa and B16–F10 cells | HSV-1 KOS | EC_50_ = 152 ng/ml | No mention | Neutralization of the virus and inhibition of binding | (Tian et al., 2022) |
| Hu2c | Vero cells; Female BALB/c mice | ACV-resistant HSV-1 | Data was not shown. | No mention | Stop the viral cell-to-cell transmission | (Bauer et al., 2017) |
| HDIT101 and HDIT102 | HEK293-6E, HEK293T and Vero cells | HSV-1 F | Virus-induced CPE of HSV-1 by 50% at a concentration of 18 nM | No mention | Bind to different epitopes of gB to blocking viral transmission | (Seyfizadeh et al., 2024) |
| Carbohydrate | | | | | | |
| L-psicose | CV-1 cell | HSV-1 KOS-GFP | EC_50_ = 99.5 mM | No mention | Inhibition of adsorption | (Muniruzzaman et al., 2016) |
| Carrageenan sulfate polysaccharides (CRGs) | Vero cells | HSV-1 L2 | CC_50_ >2000 μg/mL (CRGs), CC_50_ >2000 μg/mL (ACV); EC_50_ of 56.4~135.0 μg/mL (CRGs), EC_50_ = 2.1 ± 0.3 μg/mL (ACV) | SI = 14.8~35.7 (CRGs). SI >950 (ACV). | Inhibition of binding and penetration | (Krylova et al., 2022) |

CC_50_: 50% cytotoxic concentration;

EC_50_: 50% effective concentration;

IC50: 50% inhibitory concentration;

SI: Selectivity index, determined by the ratio of CC_50_ to EC_50_ (CC_50_/EC_50_);

MIC: Minimum inhibitory concentration;

TCID_50:_ 50% tissue culture infectious dose;

LD_50_: The median lethal dose;

MNTD: the maximum non-toxic dose.
